# Comparative analysis of metabolome and transcriptomes to explore the inhibited influence of sonodynamic therapy combined with lonidamine on hepatocellular carcinoma

**DOI:** 10.1080/10717544.2025.2593600

**Published:** 2025-12-03

**Authors:** Haoyan Tan, Huimin Tian, Yichi Chen, Bolin Wu, Wen Cheng, Haitao Shang

**Affiliations:** a Department of Ultrasound, Harbin Medical University Cancer Hospital, Harbin, People's Republic of China; b Heilongjiang Province Key Laboratory of Research on Molecular Targeted Anti-Tumor Drugs, Department of Biopharmaceutical Sciences, College of Pharmacy, Harbin Medical University, Harbin, People's Republic of China

**Keywords:** Hepatocellular carcinoma, sonodynamic therapy, lonidamine, metabolome, transcriptomes

## Abstract

Sonodynamic therapy (SDT) has emerged as a promising approach for treating hepatocellular carcinoma (HCC) by combining sonosensitizers with low-intensity ultrasound. However, SDT alone could not achieve satisfactory results. Here, we developed novel nanobubbles loaded with hematoporphyrin monomethyl ether (HMME@NBs) and evaluated its therapeutic potential in combination with the glycolytic inhibitor lonidamine (LND) against HCC. The HMME@NBs was successfully prepared with particle size of 410.58 ± 20.07 nm and zeta potential at −8.38 ± 1.12mV. The encapsulation efficiency and loading efficiency of HMME was 80.6% and 7.12%, respectively. Both *in vitro* and *in vivo* studies demonstrated that while SDT and LND monotherapies inhibited the growth of HepG2 cells and xenograft tumors in nude mice, the combination therapy exhibited the most significant inhibitory effect. Multi-omics analysis of tumor tissues revealed substantial alterations in metabolites and gene expression, with key pathways such as glutathione metabolism implicated in the treatment response. Our findings highlight the enhanced antitumor efficacy of HMME@NBs-mediated SDT combined with LND, supported by mechanistic insights from transcriptomic and metabolomic profiling. This synergistic strategy holds great potential for HCC treatment.

## 1. Introduction

Cancer has been one of the biggest threats to human health globally (Siegel et al. 2024). Liver cancer has been widely concerned because of its high mortality and poor prognosis. Hepatocellular carcinoma (HCC) is one type of liver cancer, which accounts for the majority of all liver cancer fatalities. However, the single method-based treatment strategy often proves inadequate for all patients with HCC (Kuroda et al. 2022), which has led to research on new methods or combination strategies. Consequently, emerging modalities such as photodynamic therapy (PDT) and sonodynamic therapy (SDT) have garnered increasing attention (Miller et al. 2015).

SDT, based on the synergistic effects of ultrasound irradiation and sonosensitizers, is one of the promising methods for treating various cancers (Wang et al. 2013). SDT kills tumor cells by activating accumulated sonosensitizers with ultrasound. This activation triggers electronic transitions and energy state changes, which in turn generate large amounts of reactive oxygen species (ROS) that disrupt cellular and organelle membrane integrity. SDT has many advantages compared with PDT, such as better penetrability that can efficiently target tumor tissue (Bartal et al. 2011; Wang et al. 2015). Moreover, the energy of ultrasound irradiation can be focused solely on the target tumor tissue, reducing damage to normal tissue (Gao et al. 2013). Notably, sonosensitizers as the core components of SDT critically determine therapeutic efficacy through their ROS generation capacity.

Hematoporphyrin monomethyl ether (HMME) is a traditional photosensitizer for PDT with a promising clinical application prospect (Lei et al. 2012). HMME has been widely applied in SDT due to its high yield of ROS (Zhang et al. 2017). However, HMME comprises easy-to-form aggregates due to its hydrophobic properties (Wang et al. 2022). Recently, drug delivery systems with nanoparticles have been widely researched for the treatment of cancer (Wang et al. 2016). The nanoparticles combined with sonosensitizer can achieve passive tumor targeting due to the enhanced permeability and retention (EPR) effect present in solid tumors, which has a significant application value (Bulbake et al. 2017). Nanobubbles represent promising drug delivery vehicles utilizing ultrasound-targeted microbubble destruction (UTMD) (Chen et al. 2022). Functioning as cavitation nuclei, they undergo expansion, compression, and collapse under ultrasound irradiation. This cavitation process generates microjets and shear stress, creating transient and reversible pores in cell membranes. This enhanced permeability facilitates cellular drug uptake without compromising cell viability (Tian et al. 2020). We previously synthesized sonosensitizer-loaded nanobubbles for SDT application against HCC, ​​with​​ favorable outcomes (Tan et al. 2021). Many studies have confirmed the inhibitory effect of SDT on the growth of various cancers (Li et al. 2014). However, different scholars believe that the efficacy of SDT alone was not satisfactory and advise exploring the effect of the combination of SDT with other therapeutic methods (Rosenthal et al. 2004).

Lonidamine (LND), a glycolysis inhibitor, suppresses cancer cell energy metabolism. It enhances tumor cell sensitivity by disrupting mitochondrial structure and respiration, inducing ROS generation, and promoting apoptosis (Huang et al. 2020). Critically, LND sensitizes cells to PDT, as previously demonstrated (Golding et al. 2013). SDT similarly induces mitochondrial damage, inactivates glycolytic enzymes, and depletes intracellular ATP to trigger tumor cell apoptosis (Ji et al. 2010). LND's role as an energy metabolism blocker provides a strong mechanistic rationale for its synergistic enhancement of SDT efficacy. Therefore, we propose combining LND with SDT to inhibit HCC growth.

Transcriptomics provides critical insights into gene expression patterns at the RNA level, advancing our understanding of fundamental biological processes and disease diagnostics (Zhao et al. 2021). However, its standalone application faces a fundamental limitation of the inability to directly establish causal links between transcriptional regulation and functional metabolic phenotypes. Integrated transcriptomic-metabolomic analysis overcomes this constraint, delivering unique mechanistic perspectives on metabolic reprogramming in HCC that transcend conventional single-layer approaches (Lo Re et al. 2018). This approach not only reveals the interacting biomolecular networks that govern tumor phenotypes but also identifies biomarkers validated across omics layers, thereby directly bridging transcriptional reprogramming with metabolic dysregulation.

Our prior research established the efficacy of SDT in multiple HCC cell lines, with preliminary mechanisms revealed through *in vitro* transcriptomics (Shang et al. 2021). In this study, we advanced these findings by preparing HMME-conjugated nanobubbles and combining them with LND for SDT. The safety and therapeutic effects of this combination ​​were evaluated​​ in an *in vivo* HCC xenograft model established in BALB/c nude mice. Furthermore, integrated metabolomic and transcriptomic analysis were employed to ​​elucidate​​ the mechanisms of SDT combined with LND against HCC. To our knowledge, few studies have reported the application of integrated transcriptomic and metabolomic analysis to elucidate the mechanisms of nanobubble-mediated SDT in conjunction with chemotherapy in HCC xenograft models.

## 2. Materials and methods

### 2.1. Preparation and characterization of HMME@NBs

**Scheme 1.**
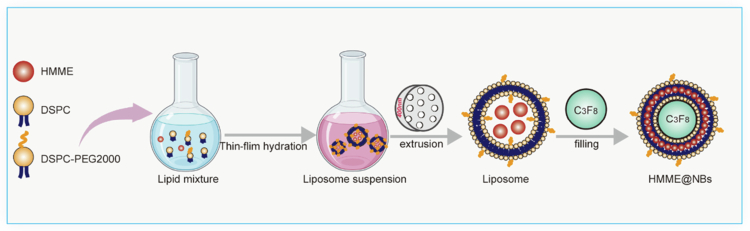
Schematic illustration of the synthesis process and structure of HMME@NBs.

As previously described, HMME@NBs were prepared using a thin-film hydration-sonication method (Scheme 1). A lipid mixture of DSPC, DSPE-PEG₂₀₀₀ (Avanti Polar Lipids, USA), and HMME powder (Macklin, China) at a mass ratio of 7:2:1 was dissolved in a solution of methanol and methylene chloride (2:1, v/v). The mixture was evaporated to dryness under reduced pressure in a round-bottom flask using a rotary evaporator, yielding a thin lipid film. This film was rehydrated in phosphate-buffered saline (PBS) within a water bath at 45–50 °C to form a lipid suspension. The suspension was extruded 15 times through a 400-nm polycarbonate membrane using a mini-extruder (Avanti Polar Lipids, USA). The resulting homogeneous liposomal suspension was transferred to a sealed plastic vial. Air within the vial was displaced by gently flushing with perfluoropropane gas (C₃F₈, Research Institute of Physical and Chemical Engineering of Nuclear Industry, China) using a three-way syringe. The sealed plastic vial was mechanically vibrated at 3800 rpm for 60 seconds (s) in a dental amalgamator (YJT Medical Apparatuses and Instruments, China). The resulting nanobubbles were then resuspended in 2 mL of sterile PBS to yield HMME@NBs. The same procedure was used to prepare pure nanobubbles (NBs) without HMME.

The morphology, distribution and particle size of HMME@NBs were observed by optical microscope (Nikon, Japan), confocal laser scanning microscope (CLSM, ZEISS LSM980, Germany) and transmission electron microscopy (TEM, Hitachi HT 7800, Japan). TEM imaging was performed at an accelerating voltage of 100 kV without the use of any specific stabilization techniques. The size distributions, polydispersity index (PDI) and zeta potentials were analyzed by dynamic light scattering (DLS, Zetasizer Nano ZS90, Malvern Instruments, UK). Samples were diluted 1000-fold with PBS prior to DLS measurement. Three consecutive measurements of 20 s each were performed per sample at 25 °C. The absorbance of pure NBs, free HMME and HMME@NBs was detected by ultraviolet-visible (UV-vis) spectrophotometer (Thermo Evolution 201, America). HMME content in HMME@NBs was measured by UV-vis spectrophotometer at 395 nm. Freshly prepared solutions of HMME at different concentrations were used to construct standard curves. The encapsulation efficiency (EE) and loading efficiency (LE) of HMME were determined using the following equation:

EE (%) = Mass of encapsulated HMME (g)/Input mass of HMME (g) × 100 (Eq1)

LE (%) = Mass of encapsulated HMME (g)/Total mass of NBs (g) × 100 (Eq2)

To assess colloidal stability, HMME@NBs were incubated in PBS at 4 °C with daily measurements of particle size and PDI over four days.

### 2.2. Cell culture

The human HCC cell line HepG2 and Huh7 were sourced from the Institute of Cancer Research at Harbin Medical University. Both cell lines were maintained in Dulbecco's Modified Eagle Medium (Hyclone, USA) supplemented with 10% fetal bovine serum (Gibco, USA) and 1% penicillin-streptomycin (Beyotime, China), and incubated at 37 °C in a humidified atmosphere containing 5% CO₂.

### 2.3. Cellular uptake test

For cellular uptake studies, PBS, free HMME and HMME@NBs were prepared. HepG2 and Huh7 cells were seeded into 35-mm confocal dishes (NEST, China) at a density of 5 × 10^5^ cells per dish and cultured for 24 hours (h). The medium was then replaced with fresh medium containing PBS, free HMME or HMME@ NBs (HMME concentration: 10 μg/mL). After 4 h incubation, cells were washed three times with PBS and stained with Hoechst 33342 (Beyotime, China) for 20 min. Following three additional washes with PBS, the cells were observed by CLSM. The fluorescence intensity of HMME was quantified by flow cytometry (BD FACSAria III, USA) according to the previous method (Zeng et al. 2013).

### 2.4. In vitro cytotoxicity and SDT capability

CCK−8 assays were performed to evaluate cytotoxicity induced by varying ultrasound (US) intensities and drug concentrations. HepG2 and Huh7 cells (1 × 10⁴/well) were seeded in 96-well plates and incubated for 24 h. For US cytotoxicity assessment, cells were exposed to different US intensities for 60 s using an ultrasonic transducer (Institute of Ultrasound Imaging, Chongqing Medical Sciences, China; 1 MHz, 50% duty cycle), followed by an additional 24 h incubation prior to viability assessment. For drug cytotoxicity assessment, the culture medium was replaced with fresh medium containing graded concentrations of LND or HMMENBs, and cell viability was assessed after 24 h incubation.

To evaluate the sonodynamic therapeutic efficacy of HMME@NBs, cells were incubated with graded concentrations of HMME@NBs for 4 h. After three PBS washes, fresh medium was added. Cells were then exposed or not to US irradiation at a fixed frequency of 1 MHz with a 50% duty cycle (1 W/cm², 60 s) and incubated for 24 h prior to viability assessment.

To investigate the synergistic effect of SDT combined with LND, cells were divided into four groups: Group A: PBS control, Group B: LND (15 μg/mL), Group C: HMME@NBs (10 μg/mL) + SDT, Group D: HMME@NBs (10 μg/mL) + SDT + LND (15 μg/mL). Cells were incubated with corresponding agents for 4 h. After three PBS washes, fresh complete medium was added. SDT groups received US irradiation (1.0 W/cm^2^, 60 s). Following 24 h incubation, cell viability was measured by CCK−8 assay.

### 2.5. Detection of intracellular ROS

HepG2 and Huh7 cells were seeded in confocal dishes and incubated for 24 h. After different treatments (with 100 μM H₂O₂ treatment for 1 h serving as the positive control), cells were washed thrice with PBS and incubated with 10 μM DCFH-DA (Beyotime, China) in serum-free medium at 37 °C for 25 min in the dark. Following three additional PBS washes, intracellular ROS levels were assessed by CLSM and quantified via flow cytometry.

### 2.6. Apoptosis assays

Cell apoptosis was assessed in HepG2 cells using an Annexin V-FITC/PI detection kit (BD Biosciences, USA) with flow cytometric analysis. Following 24 h of different treatments, cells were harvested by trypsinization, washed twice with ice-cold PBS, and resuspended in 1 × binding buffer at a density of 1 × 10^6^ cells/mL. Cell suspensions were concomitantly stained with Annexin V-FITC and propidium iodide (PI) in the dark for 15 min. Apoptosis rates were immediately quantified by flow cytometry.

### 2.7. Transwell migration assay

Cell migration assays were conducted using 12-mm diameter Transwell chambers with 3-μm polyester membranes (Corning Costar, USA). Briefly, after different treatments for 24 h, 2 × 10^4^ HepG2 or Huh7 cells were dispensed into the apical chambers with serum-free medium, while DMEM containing 10% FBS was added to the basal chambers as the chemoattractant. After incubation for 24 h, the cells migrating from apical chambers to basal side were fixed with methanol for 15 min and stained with 0.1% crystal violet.

### 2.8. Animal study

Thirty female BALB/c-nude mice aged 28–32 days and weighing 18–20 g were obtained from Vital River Laboratory Animal Technology (Beijing, China). The study employed BALB/c nude mice as the experimental animal model owing to their immunodeficiency, which enables effective establishment of tumor-bearing models using human-derived tumor cells. Additionally, female nude mice exhibit higher tolerance for cohabitation compared to males, making them more amenable to experimental handling (Ben-Ami Bartal et al. 2011). The animal studies were conducted by the ARRIVE guidelines of the Animal Care. The Experimental Animal Ethics Committee of The Fourth Affiliated Hospital of Harbin Medical University allowed animal experiments in this study. All nude mice were housed in a specific pathogen-free (SPF) facility under controlled conditions (25℃, 70 ± 5% humidity, 12-h light/dark cycle) with ad libitum access to food and water. Tumor xenografts were established via subcutaneous inoculation on the right flank using a suspension containing 1 × 10^7^ HepG2 cells in 50 μL sterile PBS, mixed at a 1:1 ratio with 50 μL Matrigel (BD Biosciences, USA). The final injection volume of 100 μL per animal was administered under brief isoflurane anesthesia (3%) to minimize procedural stress.

### 2.9. Fluorescence imaging in vivo

Among the aforementioned thirty tumor-bearing nude mice, six were selected for fluorescence imaging experiments. Following a 7-day growth period, HCC xenografts reached a mean diameter of 5 mm, at which point these six mice were enrolled in the study. HMME@NBs (10 mg/kg) were administered intravenously via the tail vein to the tumor-bearing mice under isoflurane anesthesia. Fluorescence imaging was performed at multiple time points using the Xenogen IVIS imaging system (Caliper Life Sciences, USA). At 24 h post-injection, the mice were euthanized, and major organs (heart, liver, spleen, lungs, kidneys) along with tumor tissues were harvested. *Ex vivo* fluorescence imaging of these tissues was then conducted to quantify signal intensity, enabling the assessment of HMME@NBs in vivo distribution and metabolism.

### 2.10. Treatment and measurement of tumor model

For therapeutic evaluation, the remaining 24 tumor-bearing nude mice with comparable tumor volumes were randomly assigned to four groups (*n* = 6): Group A (Control): intravenous (IV) tail vein injection of PBS. Group B (LND): intraperitoneal (IP) injection of lonidamine (15 mg/kg). Group C (SDT): IV administration of HMME@NBs (10 mg/kg), Group D (SDT + LND): combined IV administration of HMME@NBs (10 mg/kg) and IP injection of lonidamine (15 mg/kg). The *in vivo* therapeutic experiment was conducted from September 1 to 15, 2024. The LND stock solution in DMSO was stored at −80 °C in sterile, light-protected tubes. For in vivo injections, it was then diluted in normal saline (0.9% NaCl) to the desired concentration. At 4 h post-injection, groups C and D received US irradiation (2 W/cm², 60 s) targeting the tumor. Body weight and tumor dimensions (length/width) were monitored every 48 h using vernier calipers. Tumor volume (V) was calculated as: *V* = 0.523 × *L* × *W*^2^ (L: longest axis; W: perpendicular axis).

### 2.11. Safety analyses in vivo

Terminal blood sampling was performed 3 and 14 days post-intervention in all four experimental cohorts. Mice were anesthetized with 3% isoflurane (1 L/min oxygen carrier gas), followed by retro-orbital plexus puncture using heparinized 0.9-mm capillaries. Approximately 200 μL of whole blood per mouse was obtained within 10 s to minimize hemolysis. Blood analysis and biochemical parameters, including white blood cell count (WBC), red blood cell count (RBC), alanine aminotransferase (ALT), aspartate aminotransferase (AST), total bilirubin (TBIL), urea (UREA), and creatinine (CREA) were assessed using Pointcare M3 (MNCHIP, China). After euthanasia via cervical dislocation (performed under sustained isoflurane-induced anesthesia), tumor tissues and critical organs (heart, liver, spleen, lung, kidney) were rapidly harvested and fixed in 10% formalin solution. Hematoxylin and eosin (H&E) staining of primary organs from Groups B, C, and D was compared against Group A at day 3 and day 14 to assess treatment-induced injury.

### 2.12. Immunohistochemistry staining of tumor tissue

Paraffin-embedded tumor tissue sections were dewaxed, rehydrated, and blocked with 10% goat serum (10 min, RT). Sections were incubated overnight at 4 °C with anti-Ki67 antibody (1:1000; ab15580, Abcam, USA), followed by HRP-conjugated secondary antibody (1:500; ab6721, Abcam; 1 h, RT). Streptavidin-peroxidase amplification and hematoxylin counterstaining were performed. Slides were scanned using an optical microscope. Five randomly selected fields per sample were quantitatively analyzed using ImageJ software (version Fiji).

### 2.13. RNA-seq and untargeted metabolomics by liquid chromatography (LC)-MS/MS

Tumor samples harvested from xenograft nude mice in each group were collected and minced. Three samples per group were pooled in TRIzol reagent and flash-frozen in liquid nitrogen for subsequent RNA-seq analysis. RNA quantity and integrity were assessed using the RNA Nano 6000 Assay Kit on the Bioanalyzer 2100 system (Agilent Technologies, USA). Total RNA served as the input material for library preparation. Constructed libraries were initially quantified using a Qubit 2.0 Fluorometer, and insert sizes were determined with the Agilent 2100 bioanalyzer. Libraries were then pooled and sequenced on a NovaSeq 6000 (Illumina, USA). Downstream data analyses were performed using the resulting high-quality clean reads.

For LC-MS/MS analysis, six fresh samples per group were immediately flash-frozen in liquid nitrogen. Tissues (100 mg per sample) were pulverized in liquid nitrogen. The resulting homogenate was resuspended in pre-chilled methanol by vortexing, followed by centrifugation ​at 12,000 × g for 15 minutes at 4 °C​​. The supernatant from each sample was diluted with LC-MS grade water to a final methanol concentration of 53% (v/v), and centrifuged again. Finally, the supernatant was injected for LC-MS/MS analysis using a Q Exactive™ HF mass spectrometer coupled to a Vanquish UHPLC system (Thermo Fisher Scientific, Germany).

### 2.14. Western blotting assay

Western blotting was performed according to a previous study (Luo et al. 2021). Tumor tissues were lysed in prechilled RIPA buffer supplemented with phosphatase and protease inhibitors. Protein lysates were separated by SDS‒PAGE and transferred onto nitrocellulose membranes. After blocking with 5% skim milk for 2 h at room temperature, membranes were incubated at 4 °C overnight with the following primary antibodies diluted in blocking buffer: anti-Hrk (1:500; ab45419, Abcam), anti-ARC (1:500; 10846−2-AP, Proteintech, USA), and anti-GAPDH (1:2000; ab9485, Abcam) as the internal control. Membranes were washed with TBST and then incubated with HRP-conjugated goat anti-rabbit IgG (H + L) (1:10,000; ab205718, Abcam) for 2 h at room temperature. Following four TBST washes, protein bands were detected using ECL substrate (Sigma-Aldrich, USA) with exposure to X-ray film (Fujifilm, Japan) under darkroom conditions.

### 2.15. Measurement of glutathione levels

Reduced glutathione (GSH) levels in tumor tissue samples were quantified using a commercial reduced GSH colorimetric assay kit (Elabscience, USA), following the manufacturer’s protocol. Briefly, tissues were homogenized in ice-cold PBS (0.01 M, pH 7.4), centrifuged at 10,000 × g for 10 min at 4 °C, and the resulting supernatant was collected and maintained on ice for analysis. GSH concentrations were determined by measuring the optical density at 405 nm.

### 2.16. Statistical analysis

All in vitro and in vivo data are expressed as mean ± standard deviation (SD). Prior to applying one-way ANOVA, data were tested for normality using the Shapiro-Wilk test and for homogeneity of variances using Levene’s test. If both assumptions were satisfied, one-way ANOVA was performed. If either assumption was violated, non-parametric Kruskal-Wallis tests were used instead. For both parametric and non-parametric analyses, post-hoc pairwise comparisons were conducted using Tukey’s test (for all pairwise comparisons following ANOVA), respectively. All statistical tests were performed using GraphPad Prism version 8.0, with *p* < 0.05 considered statistically significant.

Differential gene expression analysis employed DESeq2 (v1.34.0), including normalization (median of ratios), dispersion estimation, negative binomial modeling, and Wald testing; *p*-values were adjusted for multiple testing using the Benjamini-Hochberg method to control the false discovery rate (FDR), yielding adjusted *p*-values (padj). Differentially expressed genes (DEGs) were defined as padj ≤ 0.05 and |log2(fold change)| ≥ 1. Batch effects (assessed by PCA/PERMANOVA) were corrected by including batch as a covariate. Functional enrichment for gene ontology (GO), Kyoto encyclopedia of genes and genomes (KEGG), and disease ontology (DO) was performed using cluster profile R package, with significance determined by hypergeometric test (padj < 0.05).

Metabolites were annotated (KEGG, HMDB, LIPIDMAPS). Differential metabolites were identified via partial least squares-discriminant analysis (PLS-DA) (VIP > 1) combined with t-test (*p* < 0.05) and |log2(fold change)| ≥ 1; univariate *p*-values were adjusted using the Benjamini-Hochberg method (FDR < 0.05).

## 3. Results

### 3.1 Preparation and characterization of HMME@NBs

**Figure 1.**
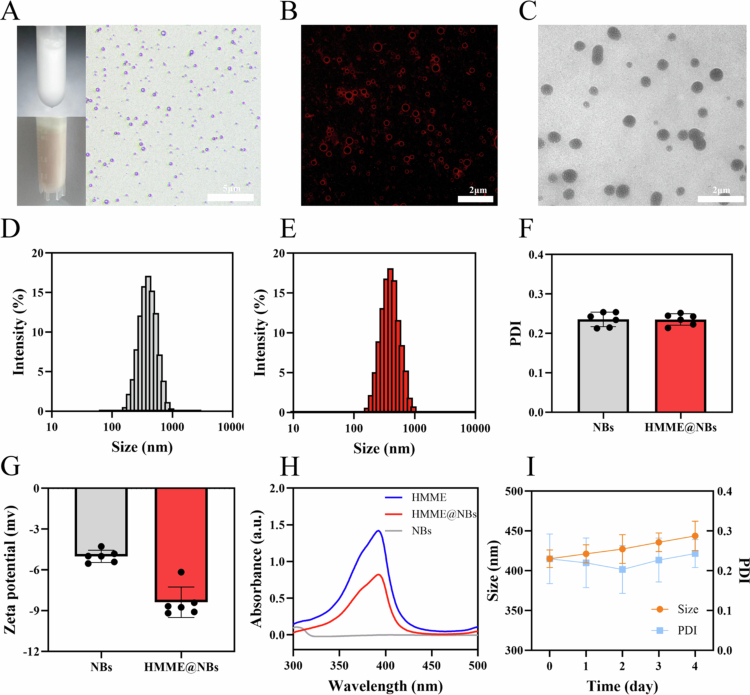
Characterization of HMME@NBs. (A) Appearance and general morphology of HMME@NBs. (B) CLSM image of HMME@NBs. (C) TEM image of HMME@NBs. (D) Size distribution of NBs. (E) Size distribution of HMME@NBs. (F) PDI of NBs and HMME@NBs. (G) Zeta potentials of NBs and HMME@NBs. (H) UV-vis spectra of HMME, NBs and HMME@NBs. (I) Szie and PDI changes of HMME@NBs in PBS solution over 4 days.

HMME@NBs were synthesized following our previously reported method (Shang et al. 2021). Visually, pure NBs formed a uniform white emulsion (Figure 1A, upper left), whereas HMME@NBs appeared as a pink emulsion (Figure 1A, lower left), confirming the successful incorporation of HMME. Optical microscopy revealed that HMME@NBs exhibited uniform distribution and consistent oval morphology (Figure 1A, right), comprising a C₃F₈ gas core surrounded by an HMME-loaded phospholipid shell. CLSM further confirmed HMME localization within the shell, as indicated by distinct red fluorescence (Figure 1B). TEM showed that HMME@NBs possessed a regular spherical structure with a smooth surface and diameters of approximately 400 nm (Figure 1C). DLS analysis yielded average hydrodynamic diameters of 395.58 ± 25.48 nm for NBs and 410.58 ± 20.07 nm for HMME@NBs (Figure 1D, E), with corresponding PDI of 0.235 ± 0.018 and 0.235 ± 0.014 (Figure 1F), respectively. Zeta potential measurements indicated that HMME loading reduced the surface charge from −5.007 ± 0.45 mV (NBs) to −8.387 ± 1.12 mV (HMME@NBs) (Figure 1G). UV-vis spectrophotometer confirmed the characteristic absorption peak of HMME within the HMME@NBs formulation (Figure 1H). The EE and LE of HMME were determined to be 80.6% and 7.12%, respectively, demonstrating efficient drug incorporation (Figure S1). Stability assessment in PBS at 4 °C showed no statistically significant changes in particle size or PDI over a 4-day period (Figure 1I), indicating satisfactory short-term colloidal stability.

### 3.2. Cellular uptake test

**Figure 2.**
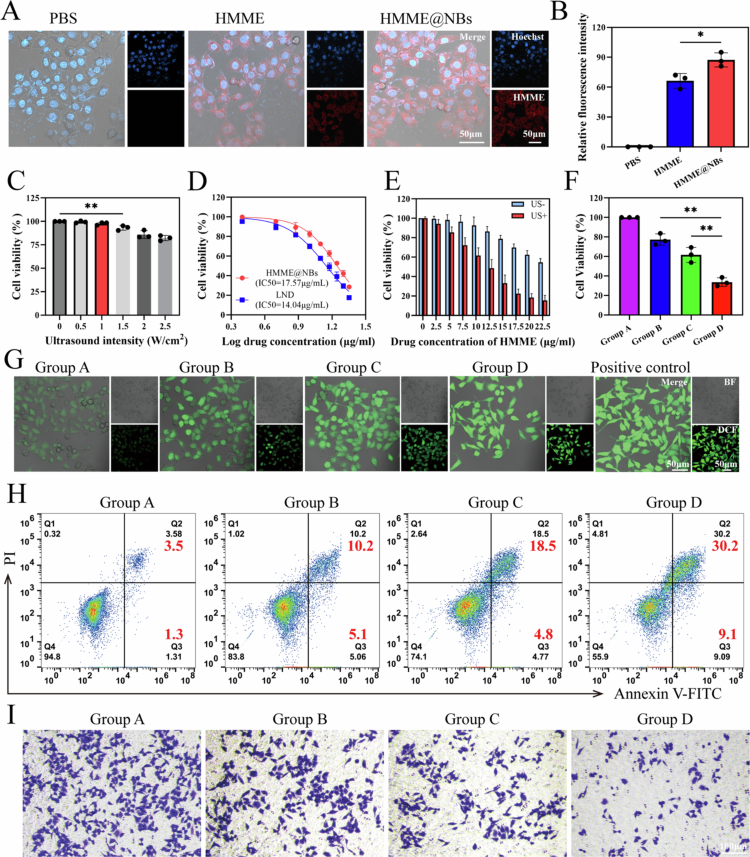
*In vitro* assay. (A) Cellular uptake of PBS, HMME and HMME@NBs in HepG2 cells using CLSM. Nuclei were stained with Hoechst 33342 (blue); HMME fluorescence is shown in red. Merge: bright-field and fluorescence overlay. (B) Quantitative analysis of the HMME fluorescence intensity in HepG2 cells. (C) Cytotoxicity of HepG2 cells treated with different ultrasound intensities. (D) Dose-effect curves of LND and HMME@C_3_F_8_-NBs in HepG2 cells. IC50 values are indicated. (E) Cytotoxicity of HMME@-NBs with or without ultrasound activation in HepG2 cells at different concentrations. (F) *In vitro* antitumor efficacy of different groups in HepG2 cells. (G) Intracellular ROS detections of HepG2 cells after different treatments by the DCFH-DA probe. ROS fluorescence is shown in green. BF: bright field. (H) Flow cytometry apoptosis analysis of HepG2 cells after different treatments. Total apoptosis rate was calculated by Q2 (early apoptosis) and Q3 (late apoptosis). (I) Representative images of HepG2 cells of lower chambers in different groups. Group A: Control group, Group B: LND, Group C: SDT, Group D: SDT + LND. Data were presented as mean ± SD (*n* = 3). *p < 0.05, **p < 0.01.

We first evaluated whether HMME@NBs could enhance the cellular internalization of HMME in HepG2 and Huh7 cells. CLSM results showed that HMME@NBs were primarily localized in the cytoplasm (Figure 2A, S2(A)). Notably, HepG2 and Huh7 cells incubated with HMME@NBs exhibited significantly stronger red fluorescence compared to those treated with free HMME, concurrently indicating enhanced cellular uptake of the drug following its encapsulation in NBs (Figure 2B, S2(B)). This fluorescence pattern suggests cellular internalization of HMME@NBs, presumably via endocytosis.

### 3.3. In vitro cytotoxicity and SDT capability

To assess cytotoxicity under varying US intensities and drug concentrations, CCK−8 assays were performed. When US intensity reached 1.5 W/cm², cell viability was significantly inhibited (Figure 2C, S3(A)). Therefore, 1 W/cm² was adopted as the standard US parameter to eliminate ultrasound-induced cytotoxicity in subsequent experiments. Both HMME@NBs and LND exhibited concentration-dependent cytotoxicity in HepG2 and Huh7 cells (Figure 2D, S3(B)). The IC50 values for HMME@NBs and LND were 17.57 and 14.04 μg/mL in HepG2 cells, and were 18.20 and 16.31 μg/mL in Huh7 cells. Guided by these results, a concentration of 15 μg/mL of LND (approximating its IC₅₀) was chosen for further in vitro evaluation.

Cytotoxicity assessment of HMME@NBs with or without US exposure demonstrated that cell viability decreased significantly under US treatment (Figure 2E, S4(A)). In both HepG2 and Huh7 cell lines, treatment with HMME@NBs (10 μg/mL HMME) followed by ultrasound-triggered SDT effectively reduced cell viability, as evidenced by a decrease from 92.78% ± 8.94% to 61.62% ± 7.96% in HepG2 cells, with a similar trend in Huh7 cells. Based on these results, this concentration was selected for further studies as it provides effective SDT outcomes without significant intrinsic cytotoxicity, thereby limiting material exposure. We therefore selected a combination of HMME@NBs (10 μg/mL HMME) for SDT and 15 μg/mL LND to evaluate their combined therapeutic effect. As shown in Figure 2F, the viability of HepG2 cells in Group D (SDT + LND) was 33.71 ± 4.41%, significantly lower than that in Group B (LND alone, 77.23 ± 5.88%) and Group C (SDT alone, 61.62 ± 7.96%). Similarly, in Huh7 cells, the viability in Group D was 39.37 ± 6.36%, also significantly lower than that in Group B (79.12 ± 5.40%) and Group C (67.39 ± 6.03%) (Figure S4(B)). These results demonstrate the efficacy of the combined LND and SDT therapy. This enhanced therapeutic effect was further confirmed by apoptosis assays (Figure 2H), which revealed cell death trends consistent with the viability results (Figure S5).

### 3.4. Detection of intracellular ROS

We simultaneously employed CLSM and flow cytometry to quantify ROS production. As shown in Figure 2G and Figure S6, distinct green fluorescence signals were observed in Groups B and C, with the most intense fluorescence appearing in Group D, indicating robust ROS generation. Quantitative analysis of fluorescence intensity corroborated these imaging findings (Figure S7). These results suggest that the enhanced effect between LND and SDT likely occurs through ROS-mediated oxidative stress damage.

### 3.5. Transwell migration assay

Transwell migration assay was conducted to evaluate the metastatic potential of HepG2 and Huh7 cells under different treatments. As shown in Figure 2I and Figure S8, Group D exhibited the fewest migrated cells among all groups (Figure S9). These results demonstrate that the combination of SDT and LND potently suppresses tumor metastasis.

### 3.6. Fluorescence imaging ability of HMME@NBs in vivo

**Figure 3.**
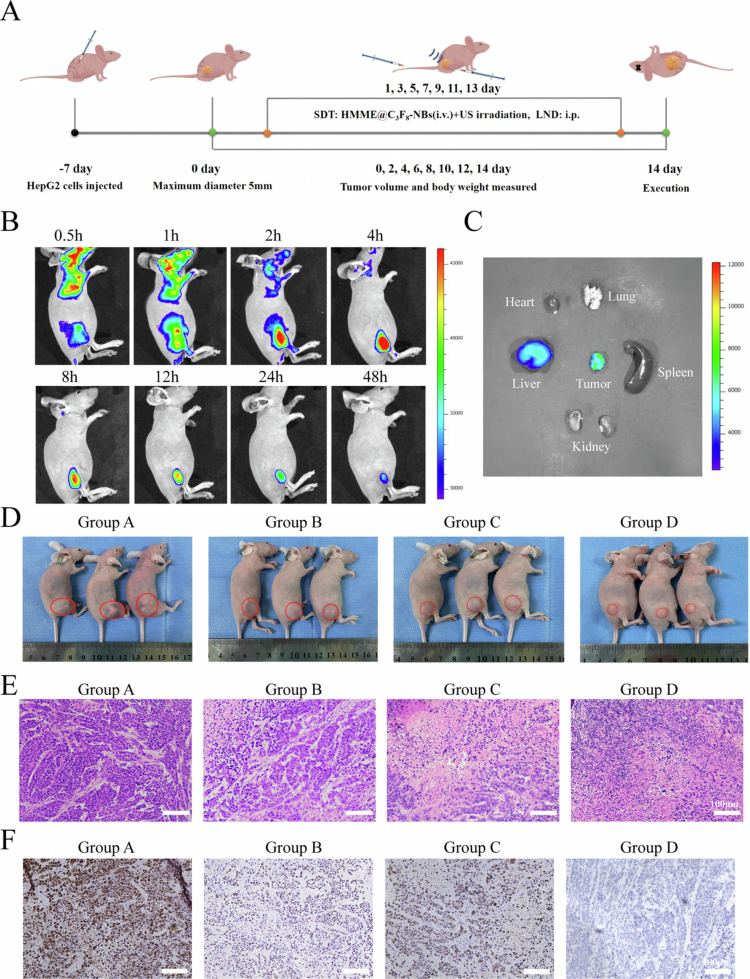
*In vivo* anti-tumor research. (A) The animal experiment flow chart. (B) *In vivo* fluorescence images of HCC tumor-bearing mice at different time points after intravenous injection of HMME@NBs. (C) *Ex vivo* fluorescence imaging of the tumor and major organs (heart, liver, spleen, lung, kidney) at 24 h post-injection. (D) Tumor volume in nude mice after different treatments over 14 days. (E) H&E staining of the tumor tissues after different treatments. (F) IHC staining images of Ki−67 expression in different groups. Group A: Control group, Group B: LND, Group C: SDT, Group D: SDT + LND. Data were presented as mean ± SD (*n* = 3). **p < 0.01.

Fluorescence imaging was employed to evaluate the in vivo distribution of HMME@NBs in tumor-bearing nude mice. As shown in Figure 3B, tumor fluorescence intensity peaked at 4 h post-injection, persisted for up to 48 h, and gradually decreased thereafter (Figure S10(A)). Based on this kinetic profile, ultrasound irradiation was applied at 4 h to maximize SDT efficacy. To further investigate biodistribution and metabolism, we quantified fluorescence in major organs and tumor tissues harvested 24 h post-injection (Figure 3C). The result reveals significantly elevated fluorescence in tumor and liver tissues (Figure S10(B)), indicating effective tumor accumulation of HMME@NBs with hepatic clearance as the primary elimination route.

### 3.7. SDT combined with LND significantly inhibited HCC tumor growth

An HCC xenograft model was established via subcutaneous HepG2 cell injection (Day −7) to assess SDT + LND anti-tumor efficacy, with the experimental timeline detailed in Figure 3A. Tumor growth was significantly suppressed in Groups B, C, and D compared to Group A following treatment initiation. Tumor volume differences among groups are detailed in Figure 3D, with Group D (SDT + LND) exhibiting the most pronounced inhibitory effect (Figure S11(A)). Upon tumor excision at Day 14, H&E staining revealed a markedly higher number of apoptotic/dead cells in Group D compared to the other groups (Figure 3E). Furthermore, the expression level of the proliferation marker Ki67 was significantly lower in Group D than in all other groups (Figure 3F, S11(B)). Collectively, these results demonstrate that SDT and LND synergistically suppress HCC through dual mechanisms: induction of tumor cell apoptosis and suppression of proliferation.

### 3.8. SDT combined with LND did not induce acute or delayed damage to animal

H&E staining of major organs was performed to evaluate the safety profile of SDT combined with LND in nude mice. Figure S12 demonstrates no significant pathological alterations in heart, liver, spleen, lung, or kidney tissues across treatment groups at day 3 and 14, with zero mortality during the 14-day experimental period. Figure S13 further indicates no statistically significant differences in hematological parameters (WBC, RBC) and serum biochemistry (ALT, AST, TBIL, UREA, CREA). Consistent with these findings, longitudinal body weight monitoring revealed no significant fluctuations at any timepoint (Figure S14), collectively confirming the absence of detectable treatment-related toxicity.

### 3.9. RNA-seq analysis in HCC xenograft tissue treated with different methods

**Figure 4.**
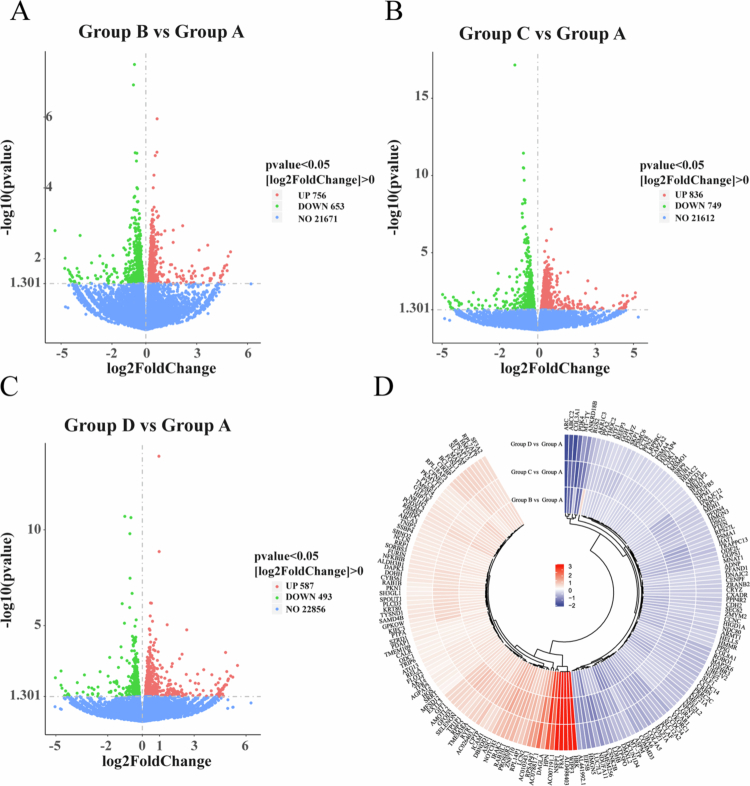
Transcriptomic analysis of tumor tissue treated using different methods. (A-C) Volcano plots displayed the differentially expressed genes (DEGs) in Group B, Group C, and Group D compared with Group A. Each red dot denotes an individually up-regulated transcript, and each green dot denotes an individually down-regulated transcript. (D) The heat map displayed the DEGs based on the integrative analysis of all treatment groups. Group A: Control group, Group B: LND, Group C: SDT, Group D: SDT + LND.

**Figure 5.**
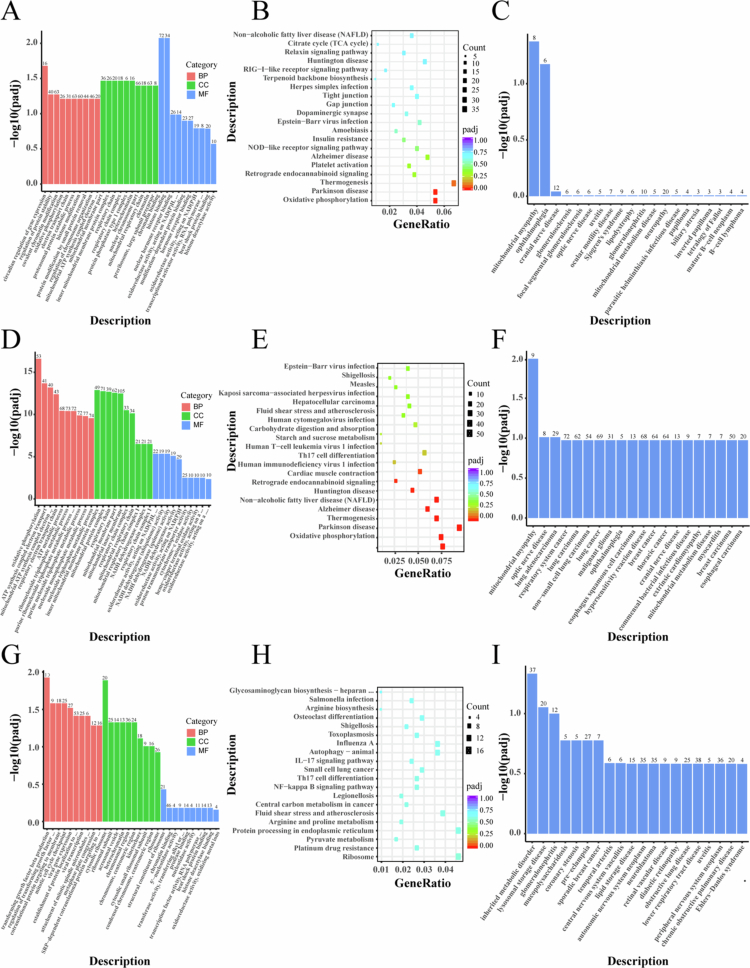
Functional enrichment analysis of differentially expressed genes. (A–C) GO, KEGG, and DO enrichment analyses of DEGs in Group B vs Group A. (D–F) GO, KEGG, and DO enrichment analyses of DEGs in Group C vs Group A. (G–I) GO, KEGG, and DO enrichment analyses of DEGs in Group D vs Group A. Group A: Control group, Group B: LND, Group C: SDT, Group D: SDT + LND.

The results revealed significant differences in mRNA expression between the treatment groups (Groups B, C, D) and the control group (Group A). Compared to Group A, Group B (LND) exhibited 756 significantly upregulated and 653 downregulated genes (Figure 4A); Group C (SDT) exhibited 836 significantly upregulated and 749 downregulated genes (Figure 4B); and Group D (SDT + LND) exhibited 587 significantly upregulated and 493 downregulated genes (Figure 4C). Analysis identified 194 DEGs common to all three treatment groups compared to the control. Subsequent heatmap analysis of these common DEGs based on their log2FoldChange values (Figure 4D) highlighted significant upregulation of genes including *HRK*, *WIPF3*, *AL049840.3*, *F8A2*, and *NSFP1*; conversely, genes such as *ARC*, *ABCC2*, *COL3A1*, *PDK4*, and *MT-TY* were significantly downregulated. Subsequent GO, KEGG, and DO enrichment analyses were conducted to elucidate the functional roles of the DEGs between groups. For Group B vs Group A, DEGs showed significant enrichment in multiple GO categories: ‘*circadian regulation of gene expression*’ and ‘*oxidative phosphorylation*’ (Biological Process), ‘*inner mitochondrial membrane protein complex*’ (Cellular Component), and ‘*phosphoprotein phosphatase activity*’ (Molecular Function) (Figure 5A); KEGG analysis revealed enrichment in ‘*Hepatocellular carcinoma*’ (Figure 5B); and DO analysis indicated enrichment for various neoplastic diseases (Figure 5C). In Group C vs Group A, DEGs were primarily enriched in mitochondrial-related GO pathways such as ‘*mitochondrial ATP synthesis coupled electron transport*’ (Figure 5D); KEGG analysis demonstrated significant enrichment for both ‘*Oxidative phosphorylation*’ and ‘*Hepatocellular carcinoma*’ (Figure 5E), while DO enrichment results are presented in Figure 5F. Notably, the enrichment patterns shifted for Group D vs Group A: GO analysis identified terms including ‘*transforming growth factor beta production*’ (Biological Process) and ‘*structural constituent of ribosome*’ (Molecular Function) (Figure 5G); KEGG analysis showed enrichment in the ‘*NF-kappa B signaling pathway*’ and ‘*IL−17 signaling pathway*’ (Figure 5H); and DO analysis again indicated enrichment for various neoplastic diseases (Figure 5I).

### 3.10. Metabolomics analysis in HCC xenograft tissue treated with different methods

**Figure 6.**
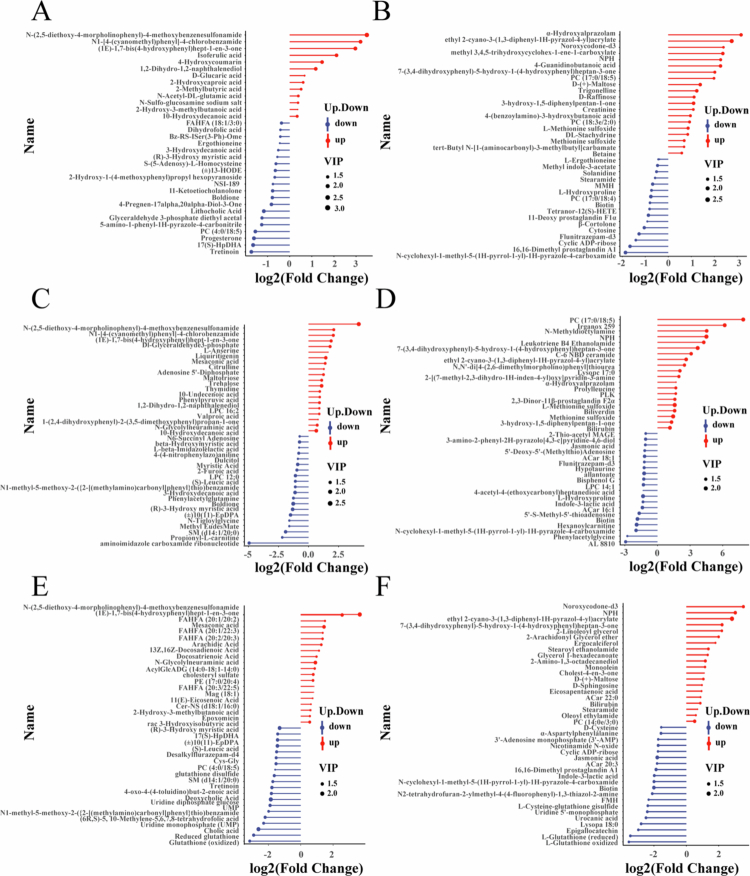
Metabolomic analysis of tumor tissue treated with different methods. (A, B) Matchstick plots depicting NEG and POS differential metabolites in Group B vs Group A. (C, D) Matchstick plots depicting NEG and POS differential metabolites in Group C vs Group A. (E, F) Matchstick plots depicting NEG and POS differential metabolites in Group D vs Group A. (Red: upregulated; Blue: downregulated). Group A: Control group, Group B: LND, Group C: SDT, Group D: SDT + LND.

**Figure 7.**
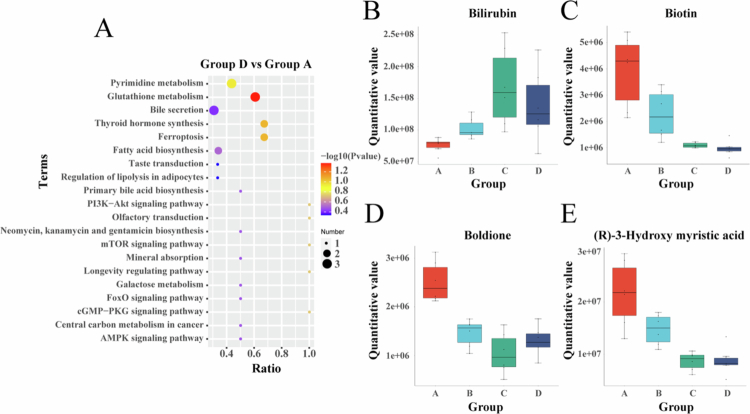
Enrichment analysis of differential metabolites. (A) KEGG pathway enrichment analysis of differential metabolites in Group D vs Group A. (B–E) Abundance changes of representative differential metabolites, shown in box plots. Group A: Control group, Group B: LND, Group C: SDT, Group D: SDT + LND.

Metabolomics analysis of HCC xenograft tissues across treatment groups revealed significant alterations. Compared to Group A, Group B exhibited 33 significantly differential metabolites in negative ion mode (NEG: 13 upregulated, 20 downregulated) and 40 in positive ion mode (POS: 24 upregulated, 16 downregulated). Group C showed 61 differential NEG metabolites (23 upregulated, 38 downregulated) and 100 differential POS metabolites (28 upregulated, 72 downregulated). Notably, Group D demonstrated the most substantial changes, with 82 differential NEG metabolites (23 upregulated, 59 downregulated) and 164 differential POS metabolites (21 upregulated, 143 downregulated). Matchstick plots visualized the direction and fold changes of these differential metabolites for Groups B (Figure 6A, B), C (Figure 6C, D), and D (Figure 6E, F) versus Group A. KEGG enrichment analysis of Group D's differential metabolites identified ‘*Glutathione metabolism*’ as significantly enriched (Figure 7A). Fourteen metabolites, including bilirubin, boldione, biotin, and (R)-3-Hydroxy myristic acid, displayed consistent directional changes across all treatment groups versus control. Box plots illustrated their expression patterns (Figure 7B–E): Bilirubin was upregulated post-treatment, most prominently in Group C; conversely, boldione, biotin, and (R)-3-Hydroxy myristic acid were downregulated, with biotin and (R)-3-Hydroxy myristic acid showing the greatest suppression in Group D, while boldione reduction was most pronounced in Group C.

### 3.11. Integration analysis of transcriptomics and metabolomic

**Figure 8.**
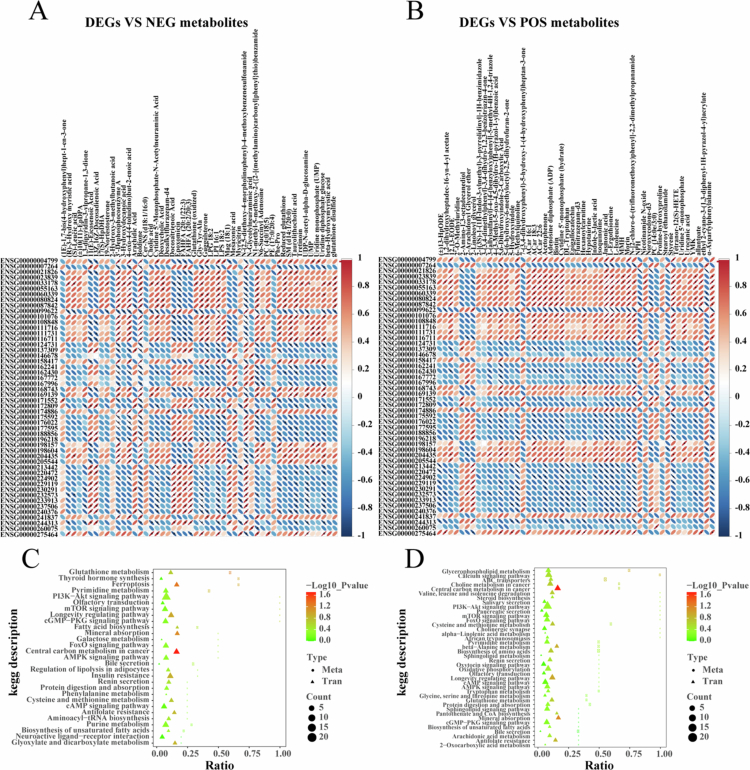
Integrated transcriptomic and metabolomic analysis. (A, B) Correlation networks between significant DEGs and differential metabolites. (C, D) KEGG pathway enrichment analysis of DEGs and differential metabolites in tumor tissue treated with SDT + LND.

In order to explore the mechanism of the therapy against HCC, integration analysis of transcriptomics and metabolomic in tumor tissue treated with SDT combined with LND in Group D was performed. Correlation analysis was conducted between the significant differential from transcriptome analysis and significant differential metabolites from metabolomics analysis to measure the degree of association between DEGs and differential metabolites (Figure 8A, B). The results showed that multiple metabolites were significantly correlated with multiple genes. For example, boldione was negatively correlated with *AC010343.1*, biotin was negatively correlated with *TMEM238*, and there was a significant positive correlation between epigallocatechin, L-Glutathione oxidized and *TBC1D3G*. Moreover, all the obtained differential genes and metabolites were mapped to the KEGG pathway database to obtain the common pathway information after treatment with SDT combined with LND (Figure 8C, D). The results show that the DEGs and differential metabolites were enriched into the ‘*Glutathione metabolism*’ and ‘*Central carbon metabolism in cancer*’ after the treatment of HCC xenograft with SDT combined with LND significantly.

### 3.12. Validate transcriptomics and metabolomic sequencing data

As previously established, transcriptomic analysis identified 194 DEGs common to all three treatment groups versus controls. Among these, *Harakiri* (HRK) expression was significantly up-regulated whereas *Apoptosis Repressor with Caspase recruitment domain* (ARC) was markedly down-regulated. To validate these findings, we performed Western blotting analysis on tumor tissues from Group A and Group D. Consistent with sequencing data, HRK protein levels were elevated in Group D relative to Group A, while ARC expression was significantly reduced (Figure S15). Furthermore, integrated multi-omics analysis demonstrated significant enrichment of both DEGs and differential metabolites in the glutathione metabolism pathway following SDT-LND combination therapy. Subsequent quantification revealed that, as anticipated, Group D exhibited significantly decreased GSH levels compared to Group A (Figure S16).

## 4. Discussion

The primary mechanisms of SDT encompass multiple pathways, including reactive ROS generation and enhanced anti-tumor immunity, with ROS generation constituting the dominant paradigm (Rengeng et al. 2017). Ultrasound irradiation of tumor tissues containing sonosensitizers induces substantial ROS production, eliciting cytotoxic effects (Hao et al. 2014). Given the decisive role of sonosensitizers in SDT efficacy, we developed HMME@NBs to overcome the aggregation tendency of free HMME caused by its hydrophobicity (Dai et al. 2014). The engineered HMME@NBs exhibit nanoscale dimensions with uniform size distribution and maintain colloidal stability in PBS for > 4 days without significant particle size changes. Leveraging the EPR effect, HMME@NBs achieve efficient peritumoral accumulation (Maeda et al. 2013), with *in vivo* fluorescence imaging experimental results confirming robust tumor enrichment and sustained intratumoral retention exceeding 48 h. More importantly, when exposed to ultrasound irradiation, the HMME@NBs selectively disintegrate within the target tumor tissue, releasing HMME. This enhances the selectivity of SDT and minimizes damage to surrounding healthy tissue (Zhang et al. 2017). However, the *in vivo* efficacy of this approach for inhibiting tumor growth remains suboptimal (Rosenthal et al. 2004).

Current evidence indicates continuous enhancement of synergistic therapeutic effects in chemo-SDT combinations, establishing this modality as a leading strategy undergoing refinement (Liang et al. 2020). LND, which exerts anti-tumor activity by disrupting energy metabolism, has been investigated in multiple cancers including breast, colon, and non-small cell lung carcinomas (Gourdier et al. 2002; Pacini et al. 2000). However, its transient and reversible effects limit monotherapeutic efficacy. Subtherapeutic doses yield inadequate responses, while escalated dosing induces significant toxicity and compromised biocompatibility (Li et al. 2013). SDT induces mitochondrial damage, inactivates glycolytic enzymes, and depletes intracellular ATP to trigger tumor cell apoptosis (Ji et al. 2010). This energy-disrupting mechanism provides a rational basis for LND—an energy metabolism inhibitor—to potentiate SDT sensitivity. In our prior research, phospholipid microbubbles co-functionalized with HMME and LND demonstrated potent *in vitro* efficacy against diverse HCC cell lines (Shang et al. 2021). Nevertheless, anti-tumor activity in human HCC xenograft models remains uncharacterized, particularly regarding transcriptomic alterations and metabolic reprogramming dynamics. To address this gap, HepG2-xenografted nude mice were employed to evaluate tumor growth inhibition.

As anticipated, both SDT and LND monotherapies inhibited HepG2 and Huh7 cells proliferation and xenograft tumor growth, with the combination therapy demonstrating the most potent inhibitory effects. Critically, the SDT-LND combination induced no acute or chronic toxicity in experimental animals, as evidenced by intact histoarchitecture of vital organs on H&E staining and unaltered serum biochemical parameters. Thus, HMME@NBs represent a promising sonosensitizer for SDT applications. Building on these findings, HMME@NBs-mediated SDT combined with chemotherapy emerges as a viable therapeutic strategy for HCC, demonstrating both efficacy and safety. To elucidate the specific molecular mechanisms underlying the SDT-chemotherapy combination, we conducted integrated transcriptomic and untargeted metabolomic analyses. These multi-omics investigations aim to identify potential biomarkers—including differentially expressed mRNAs, altered metabolic profiles, and dysregulated pathways—associated with HCC pathogenesis.

Transcriptomic analysis of DEGs is critical for exploring HCC pathogenesis. Our study identified several DEGs as potential HCC biomarkers through transcriptomic profiling. Results revealed that LND, SDT, and their combination significantly altered mRNA expression profiles in tumor tissues. Among the most prominently dysregulated genes, *HRK* —a proapoptotic Bcl−2 family member—exhibited significant upregulation across all treatment groups. *HRK* inactivation is implicated in tumorigenesis across diverse cancer types (Hao et al. 2012), while *HRK*-expressing tumors demonstrate reduced proliferative capacity, diminished vascularization, and improved survival in murine models (Kaya-Aksoy et al. 2019). Collectively, these findings establish *HRK* activation as a promoter of HCC cell apoptosis. Experimentally, both LND and SDT monotherapies induced apoptosis, with maximal effect in ​​the combination group​​. Mechanistically, ​​combination group HRK protein elevation​​ was confirmed by Western blotting.

Additionally, we observed significant downregulation of multiple post-treatment genes, most notably *ARC*. ARC, an endogenous inhibitor of apoptosis, suppresses both death receptor and mitochondrial/ER-mediated apoptotic pathways (Nam et al. 2021). *ARC* expression is markedly elevated across diverse malignancies (Mercier et al. 2008; Ziegler et al. 2008), with Ras signaling driving its upregulation through transcriptional activation of *NOL3* (Wu et al. 2010). Clinically, *ARC* overexpression serves as a predictor of tumor invasion and metastasis in human cancers (Takata et al. 2006). Moreover, prior studies have established that *ARC* promotes chemoresistance by inhibiting mitochondrial fission (Wang et al. 2009). In this work, SDT-induced *ARC* downregulation not only suppressed HCC tumor growth but may also attenuate metastasis and reverse chemoresistance. However, validating these effects requires longitudinal monitoring in animal models and evaluation using chemoresistant HCC cell lines; these investigations will be prioritized in future studies.

We found that these DEGs were enriched in multiple pathways, including ‘*oxidative phosphorylation*’, ‘*NF-κB signaling pathway*’, and ‘*IL−17 signaling pathway*’. This observation is consistent with previous studies, which also reported significant enrichment of oxidative phosphorylation and IL−17 signaling among DEGs following SDT treatment (Feng et al. 2023). Tumor cells utilize the TCA cycle and oxidative phosphorylation to generate ATP and balance ROS (DeBerardinis and Chandel 2016); tumorigenesis is dependent on mitochondrial function. Specifically, tumor cells employ fatty acids and amino acids to sustain mitochondrial respiration, particularly during alterations in the tumor microenvironment (Palm and Thompson 2017). Therefore, the enrichment of ‘*oxidative phosphorylation*’ further confirms that SDT and LND induce tumor cell apoptosis by disrupting mitochondrial functions. Additionally, both the ‘*NF-κB signaling pathway*’ and ‘*IL−17 signaling pathway*’ critically regulate tumorigenesis and progression. These transcriptomic results demonstrate that SDT and LND inhibit HCC growth through coordinated modulation of multiple genes and pathways.

Metabolites with low molecular weight collectively constitute the ‘metabolome,’ which dynamically changes in HCC (Wishart et al. 2018). Metabolomics aims to identify and measure metabolites, and discover biomarkers (Ganti and Weiss 2011). We identified several differential metabolites exhibiting similar trends in the treatment group compared to the control group; bilirubin and biotin serve as illustrative examples. The results showed that bilirubin levels were significantly upregulated, while biotin levels were downregulated. Bilirubin, an endogenous metabolic product derived from heme metabolism, is recognized as a potentially toxic compound (Cayabyab and Ramanathan 2019). However, unconjugated bilirubin can enter cells and influence apoptosis (Hankø et al. 2005). Evidence also suggests it may protect against cancer development through its potent antioxidant properties (Harpavat et al. 2016). Elevated serum unconjugated bilirubin has been associated with a reduced risk of certain cancers (Zucker et al. 2004). Collectively, these findings indicate that bilirubin may inhibit tumor cell growth, offering a novel perspective in disease biology (Gazzin et al. 2016). These established theories corroborate our results, demonstrating a significant upregulation of bilirubin in tumor tissue following SDT and LND treatment. Additionally, we observed a significant downregulation of biotin in tumor tissues post-treatment. Biotin, a water-soluble B-complex vitamin, is an essential nutrient for all living organisms (León-Del-Río 2019). It serves as an essential co-factor for carboxylases involved in fatty acid synthesis and energy production (Birnbaum and Stulc 2017) and acts as a regulator of gene expression (Maeda et al. 1996). The metabolic and gene regulatory pathways of biotin are oriented towards ensuring functional carboxylases and optimizing tissue-specific biotin utilization (León-Del-Río 2019). However, the specific relationship between biotin and liver disease development, particularly HCC, remains incompletely characterized and warrants further investigation.

Meanwhile, transcriptomics can be integrated with metabolomics for biomarker screening. Integrated analysis revealed significant correlations between multiple metabolites and multiple genes. Furthermore, both DEGs and differential metabolites were significantly enriched in the ‘*Glutathione metabolism*’ and ‘*Central carbon metabolism in cancer*’ pathways following treatment of HCC xenografts with SDT combined with LND. Within the tumor microenvironment, GSH serves as a crucial antioxidant defense mechanism, neutralizing ROS to alleviate oxidative stress (Yuan et al. 2023). Consequently, targeted GSH depletion represents a promising therapeutic approach to intensify oxidative damage in malignant cells. Previous studies indicate that LND potentiates ferroptosis through concurrent targeting of two metabolic pathways: glycolytic inhibition reduces cellular ATP generation, while pentose phosphate pathway suppression diminishes nicotinamide adenine dinucleotide phosphate biosynthesis, collectively accelerating GSH depletion (Zhang et al. 2023). This study confirmed that the combination treatment group exhibited significantly decreased GSH levels compared to the control group, which may be attributed to LND intervention. Further quantitative analysis of ROS demonstrated markedly higher fluorescence intensity in the combination treatment group than in other groups, indicating that LND effectively enhances SDT-mediated oxidative stress. These results demonstrate that LND sensitizes SDT efficacy, providing new perspectives for developing SDT-based combination treatment strategies.

This study has several limitations. First, the *in vivo* dosing regimen was guided by a pilot study; however, translating *in vitro*-optimized doses into *in vivo* settings remains a significant challenge that warrants further investigation. Second, the RNA-seq analysis was conducted with only three biological replicates per group. Despite strong intra-group consistency and the application of stringent statistical thresholds (FDR < 0.05 and |log2FC| > 1), this small sample size may reduce the statistical power of the analysis. Third, the biomarkers identified through multi-omics screening require further systematic validation by molecular biology experiments. Fourth, the abbreviated observation period in our animal model precludes a comprehensive assessment of the treatment effects on tumor metastasis and long-term survival rates. To overcome these limitations, we plan to establish an orthotopic HCC transplantation model in future work to more thoroughly investigate the regulatory mechanisms of SDT within the tumor microenvironment.

## 5. Conclusion

In summary, we successfully developed HMME@NBs, a novel sonosensitizer enabling efficient SDT combined with synergistic chemotherapy to enhance HCC treatment. Leveraging the EPR effect of nanoscale lipid bubbles, HMME@NBs selectively accumulate in tumor tissues. Upon ultrasound irradiation, substantial ROS generation effectively induces HCC cell apoptosis and, when combined with LND, significantly suppresses tumor growth in HCC models. Multi-omics analysis revealed profound alterations in gene expression and metabolic profiles, uncovering critical biomarkers and pathways for HCC precision therapy. These findings advance SDT mechanism research and provide strategic insights for future HCC treatment development.

## Supplementary Material

Supplementary Material Supplementary Materials.

## Data Availability

The datasets presented in this study can be found in online repositories. The names of the repositories and accession number(s) can be found at: https://www.ncbi.nlm.nih.gov/sra/PRJNA905344, https://www.ncbi.nlm.nih.gov/biosample/31870931, https://www.ncbi.nlm.nih.gov/biosample/31870932, https://www.ncbi.nlm.nih.gov/biosample/31870933, https://www.ncbi.nlm.nih.gov/biosample/31870934.
