# Supplementary material for: Comparative analysis of metabolome and transcriptomes to explore the inhibited influence of sonodynamic therapy combined with lonidamine on hepatocellular carcinoma
Source: Drug Deliv. 2025 Dec 3;32(1):2593600. doi: 10.1080/10717544.2025.2593600 (PMC12677030; doi:10.1080/10717544.2025.2593600)
Supplement: Supplementary Material — s. [file IDRD_A_2593600_SM2248.docx]

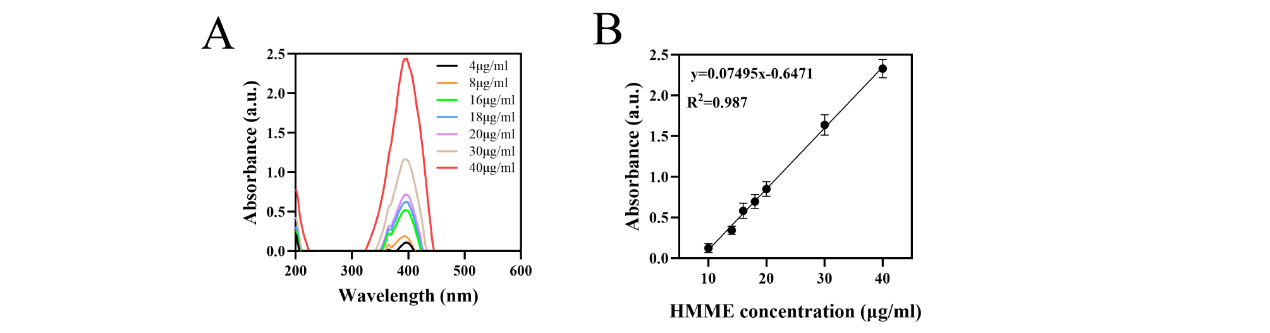


Figure S1. (A) UV-vis spectra of HMME at different concentrations; (B) The linear relationship between HMME and concentration.


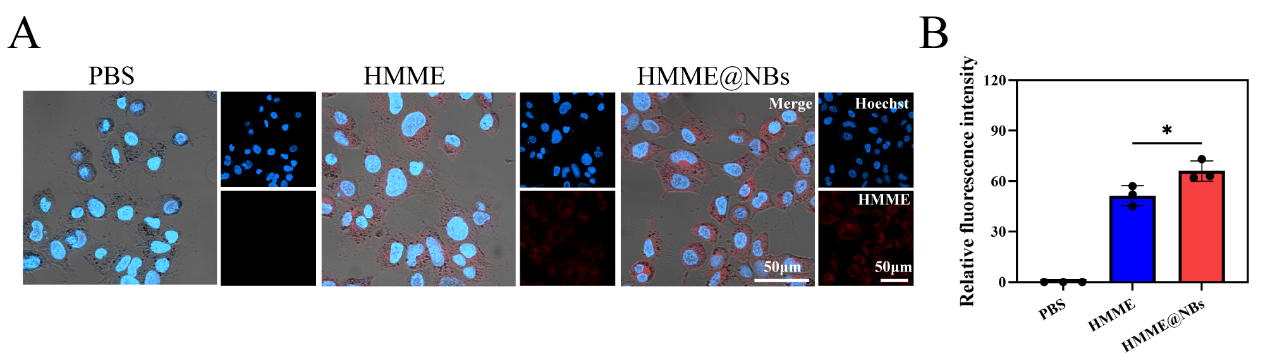


Figure S2. (A) Cellular uptake of PBS, HMME and HMME@NBs in Huh7 cells using CLSM. Nuclei were stained with Hoechst 33342 (blue); HMME fluorescence is shown in red. Merge: bright-field and fluorescence overlay. (B) Quantitative analysis of the HMME fluorescence intensity in Huh7 cells. Data were presented as mean ± SD (n = 3). *p < 0.05.


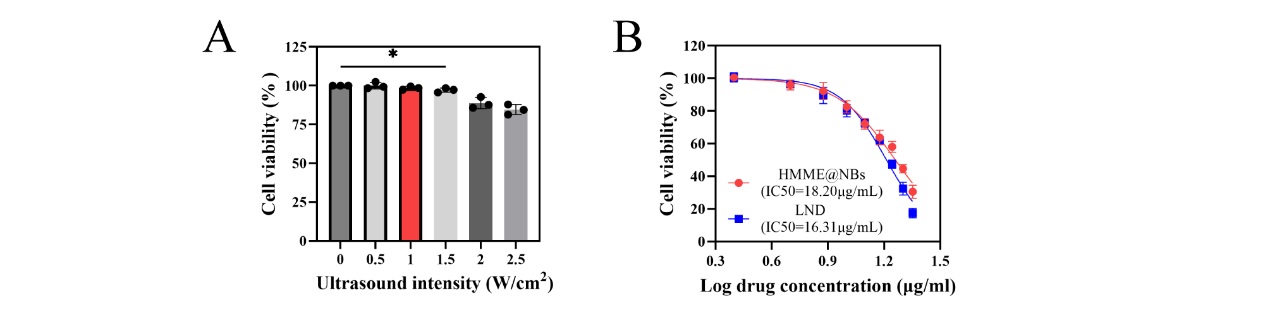


Figure S3. (A) Cytotoxicity of Huh7 cells treated with different ultrasound intensities. (B) Dose-effect curves of LND and HMME@C_3_F_8_-NBs in Huh7 cells. IC50 values are indicated. Data were presented as mean ± SD (n = 3). *p < 0.05.


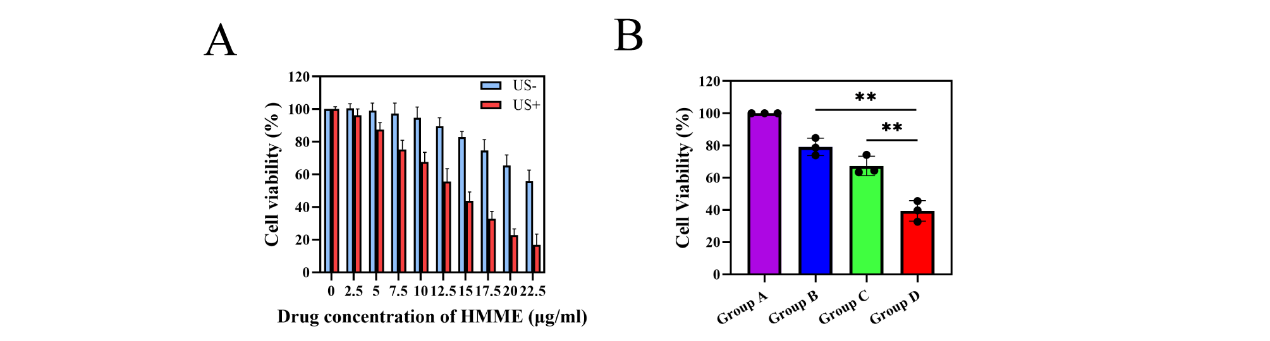


Figure S4. (A) Cytotoxicity of HMME@-NBs with or without ultrasound activation in Huh7 cells at different concentrations. (B) *In vitro* antitumor efficacy of different groups in Huh7 cells. Group A: Control group, Group B: LND, Group C: SDT, Group D: SDT + LND. Data were presented as mean ± SD (n = 3). **p < 0.01.


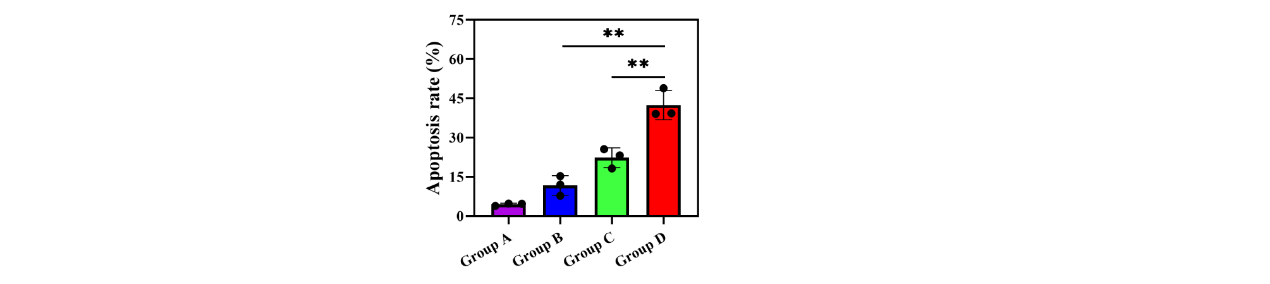


Figure S5. Quantification of total apoptosis in HepG2 cells under different treatments. Group A: Control group, Group B: LND, Group C: SDT, Group D: SDT + LND. Data were presented as mean ± SD (n = 3). **p < 0.01.


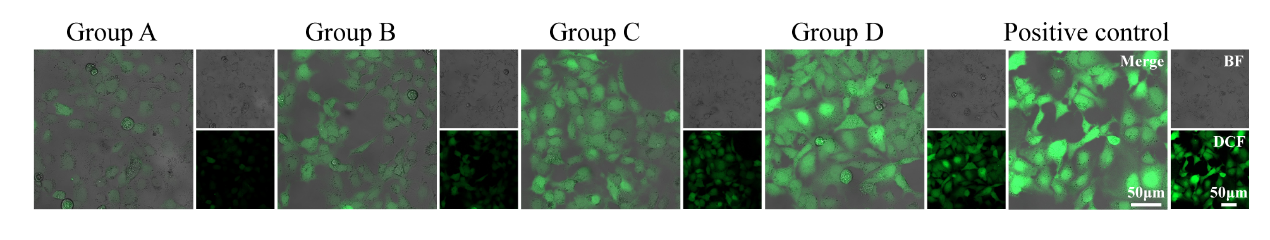


Figure S6. Intracellular ROS detections of Huh7 cells after different treatments by the DCFH-DA probe. ROS fluorescence is shown in green. BF: bright field. Group A: Control group, Group B: LND, Group C: SDT, Group D: SDT + LND.


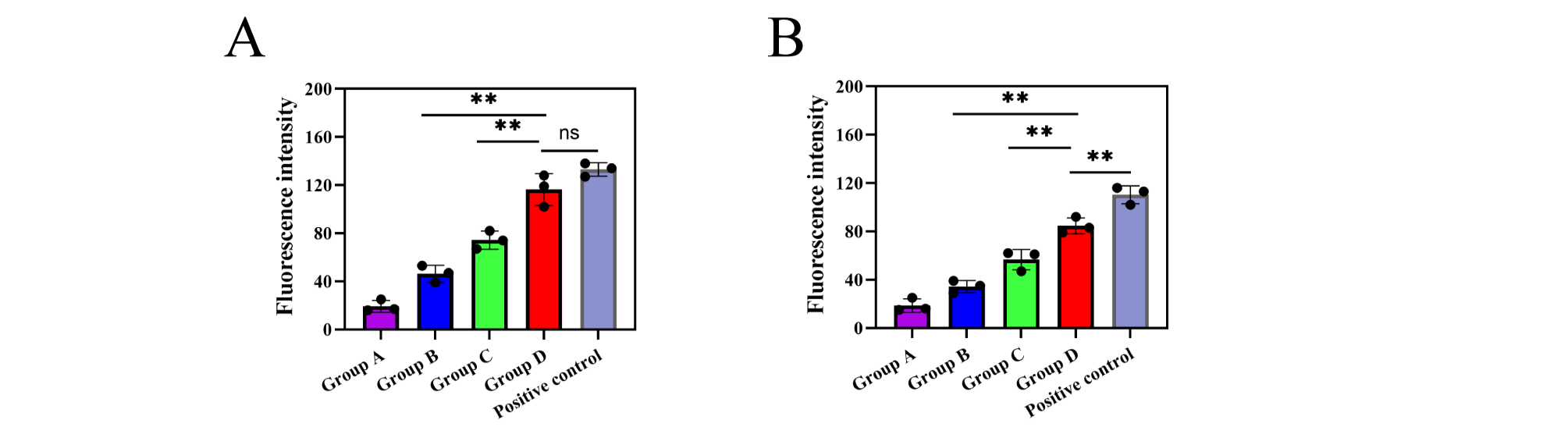


Figure S7. (A, B) Quantification of ROS levels in HepG2 and Huh7 cells under different treatments. Group A: Control group, Group B: LND, Group C: SDT, Group D: SDT + LND. Data were presented as mean ± SD (n = 3). ns: no significance, **p < 0.01.


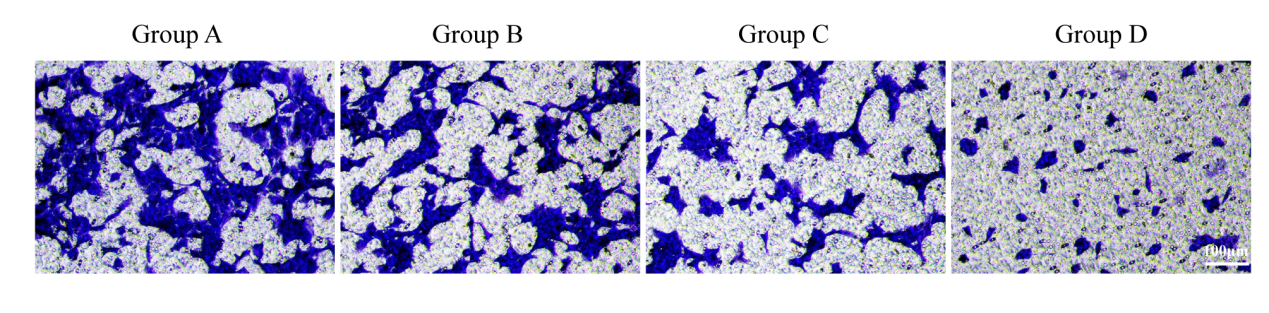


Figure S8. Representative images of migrated Huh7 cells in the lower chambers under different treatments. Group A: Control group, Group B: LND, Group C: SDT, Group D: SDT + LND.


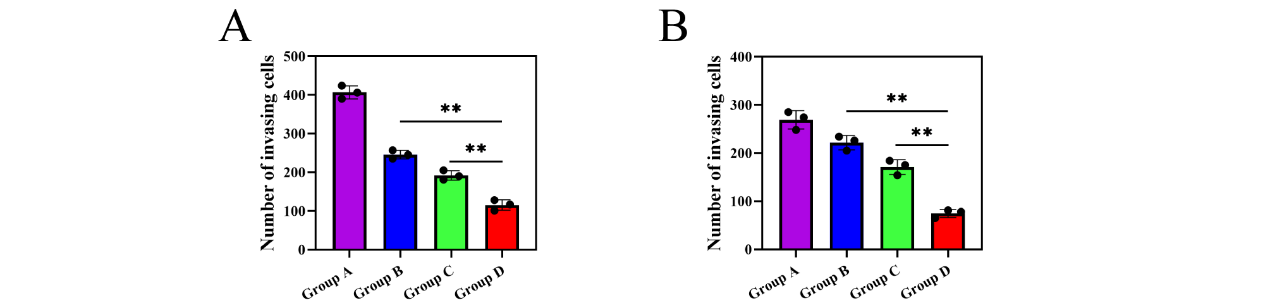


Figure S9. (A, B) Number of migrated HepG2 and Huh7 cells in the lower chamber under different treatments. Group A: Control group, Group B: LND, Group C: SDT, Group D: SDT + LND. Data were presented as mean ± SD (n = 3). **p < 0.01.


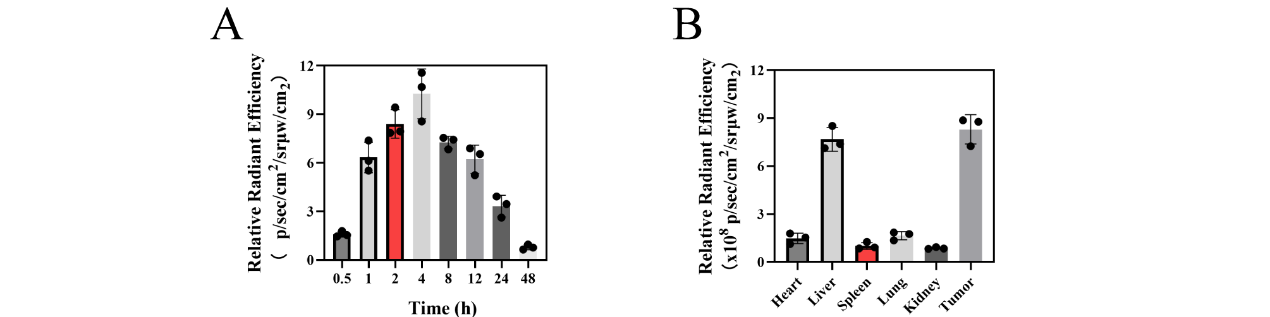


Figure S10. (A) Quantification of the mean fluorescence intensities within tumor tissues. (B) Quantitative analysis on the mean fluorescence intensity of tumor and major organs harvested from the mice with intravenous injection of HMME@NBs. Data were presented as mean ± SD (n = 3).


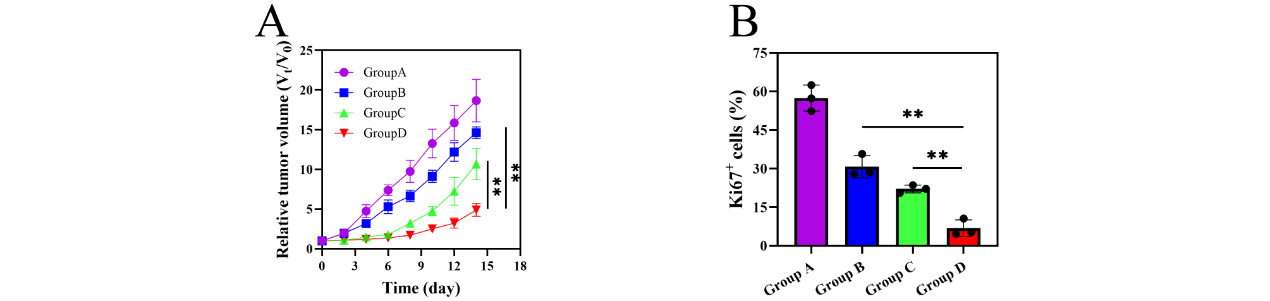


Figure S11. (A) Monitoring tumor volume in nude mice over 14 days following different treatments. (B) Ki67 positive rate among different treatment groups. Group A: Control group, Group B: LND, Group C: SDT, Group D: SDT + LND. Data were presented as mean ± SD (n = 3). **p < 0.01.


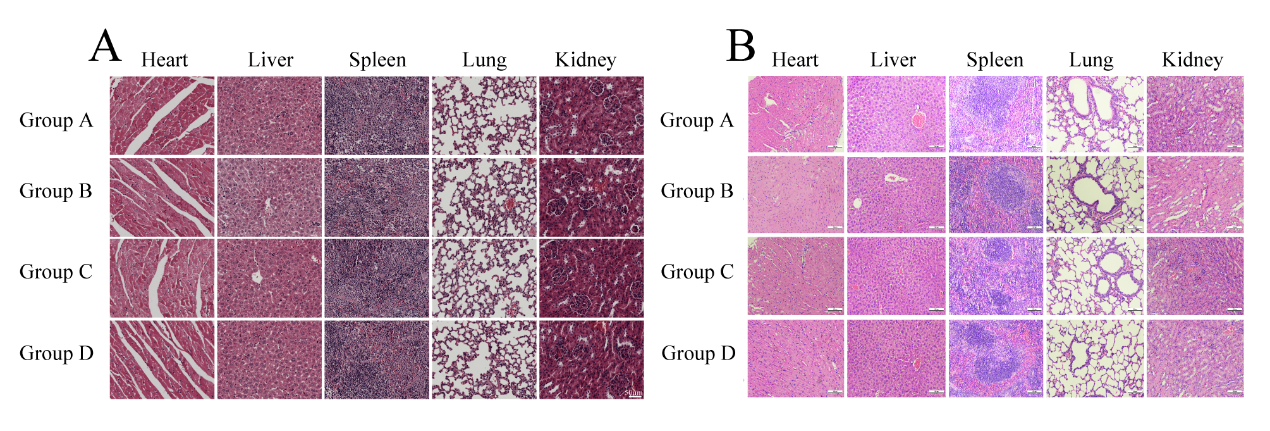


Figure S12. H&E staining of major organs from different groups (A) on day 3 and (B) on day14. Group A: Control group, Group B: LND, Group C: SDT, Group D: SDT + LND.


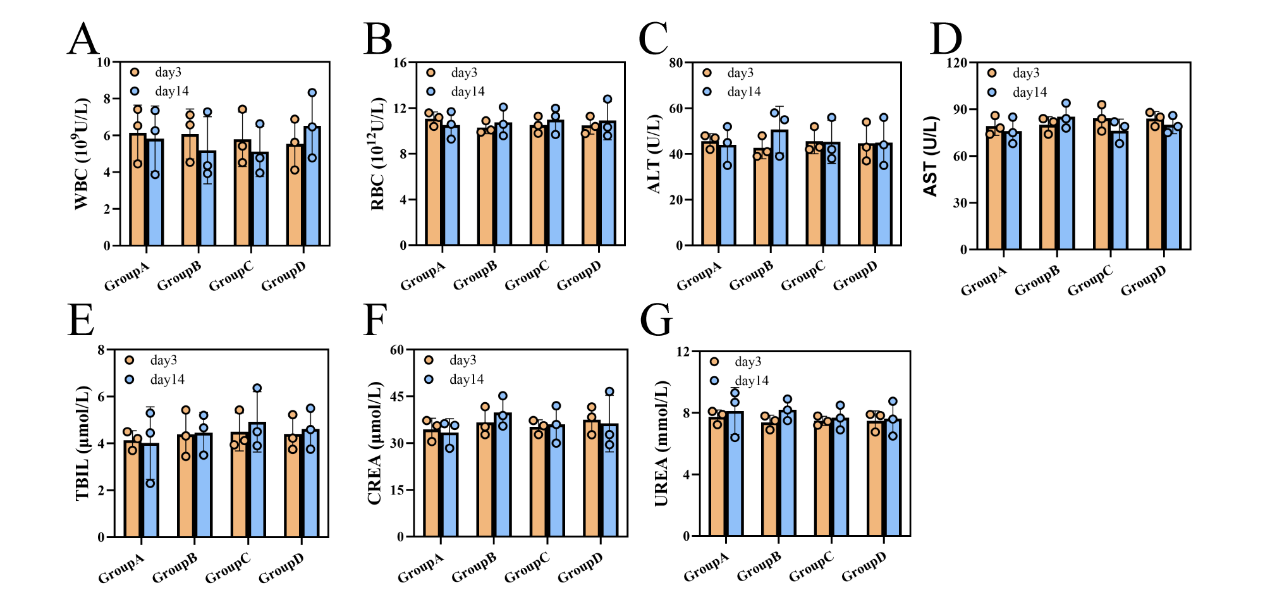


Figure S13. Changes in hematological and biochemical parameters of nude mice at day 3 and day 14 after different treatments. Measured parameters include: (A) white blood cell count (WBC), (B) red blood cell count (RBC), (C) alanine aminotransferase (ALT), (D) aspartate aminotransferase (AST), (E) total bilirubin (TBIL), (F) creatinine (CREA), and (G) urea (UREA). Group A: Control group, Group B: LND, Group C: SDT, Group D: SDT + LND. Data were presented as mean ± SD (n = 3).


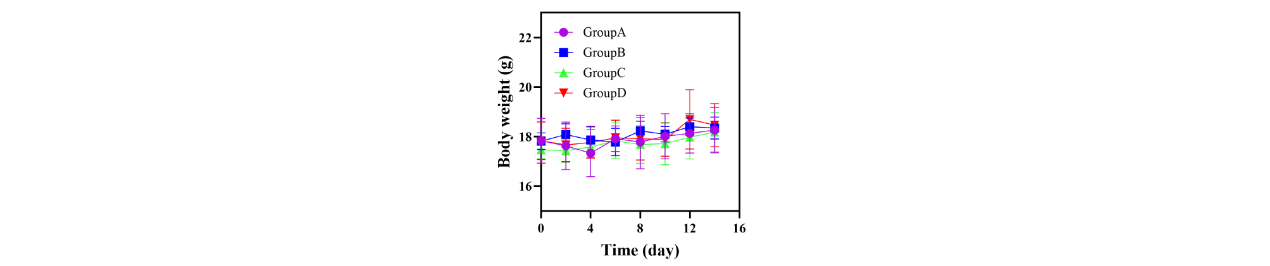


Figure S14. Monitoring body weights in nude mice over 14 days following different treatments. Group A: Control group, Group B: LND, Group C: SDT, Group D: SDT + LND. Data were presented as mean ± SD (n = 3).


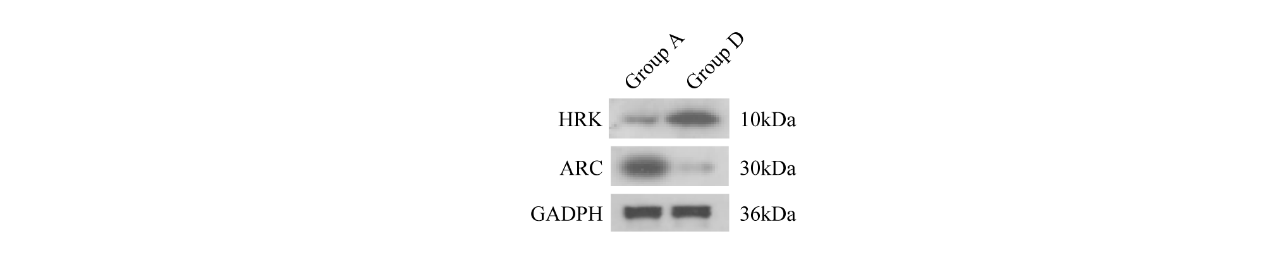


Figure S15. Western blotting analysis of HRK and ARC protein expression in Group A (Control) vs Group D (SDT + LND).


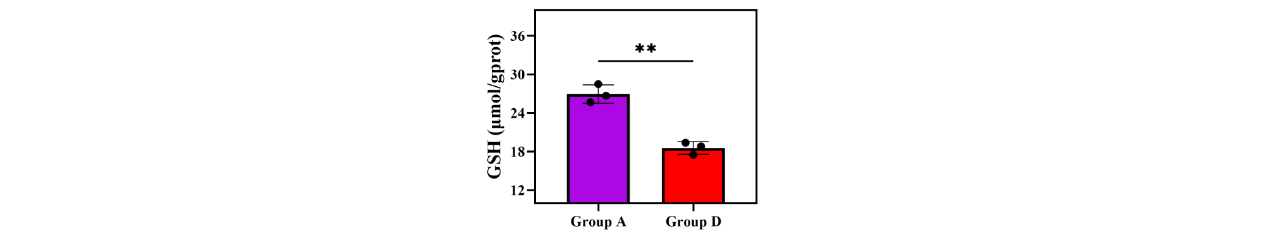


Figure S16. Tumor tissue glutathione (GSH) levels in Group A (Control) vs Group D (SDT + LND). Data were presented as mean ± SD (n = 3). **p < 0.01.
